# Effect of hydroxyethyl starch on acidosis in patients with aluminum phosphide poisoning

**DOI:** 10.22088/cjim.10.3.271

**Published:** 2019

**Authors:** Gholamabbas Kafi, Samaneh Akbarpour, Mohammad Arefi, Behnam Behnoush, Mahin Ahmadi Pishkuhi, Nasrin Barzegari

**Affiliations:** 1 Forensic Medicine and Toxicology, Tehran University of Medical Sciences, Tehran, Iran.; 2 Occupational Sleep Research Center (OSRC), Baharloo Hospital, Tehran University of Medical Sciences, Tehran, Iran; 3 Baharloo Hospital, School of Medicine, Tehran University of Medical Sciences, Tehran, Iran; 4 Pars Advanced and Minimally Invasive Medical Manners Research Center, Pars Hospital, Iran University of Medical Science, Tehran, Iran

**Keywords:** Hydroxyethyl Starch, Aluminum Phosphide, Rice tablet

## Abstract

**Background:**

Given the fact that various studies have reported the positive effects of hydroxyethyl starch therapy in controlling shock, this study aimed to compare the effects of hydroxyethyl starch on modifying acidosis and treating patients with aluminum phosphide poisoning.

**Methods:**

This was a randomized clinical trial that was conducted on 60 patients with aluminum phosphide poisoning. We compared the two groups of patients treated with hydroxyethyl starch and normal saline. Base excess and serum pH of arterial blood gases (ABG) were measured before and after the treatment and compared by t-test.

**Results:**

Results showed that arterial pH in the starch group and normal saline group increased by 0.13 and 0.18, respectively, and the difference between the two groups was not statistically significant. The difference in the base excess before and after treatment in the starch group and normal saline group was 6.41 and 5.39, respectively, and the difference between the two groups was not statistically significant. Changes in mean values of arterial pH after the intervention in comparison with before treatment were statistically significant (p<0.05).

**Conclusion:**

Overall, the results of the present study show that starch is at least as effective as normal saline in treating acidosis in patients poisoned with aluminum phosphide and can be used instead of normal saline, and both of the two treatments could be equally effective.

Aluminum phosphide (ALP) is a very toxic substance that is recognized with various trade names like Fastphos, Fumitoxin, Gastoxin, Max-Kill, Phosfume, and Phostoxin. However, in Iran, it is known as rice tablet which is used to protect stored rice from pests (1-3). Deliberate self-poisoning with rice tablet to commit a suicide is very common in Iran and its prevalence is increasing day by day. In view of that, one of the most common types of poisoning in Iran and India is rice tablet poisoning (4-6). More specifically, there is usually an increase in the number of deliberate self-poisoning with rice tablet during the growing (agricultural) season. When exposed to water or steam, the tablet produces phosphine, ammonia, and carbon dioxide gases. 

It is not absorbed through the skin and causes poisoning through oral use and respiration. The severity of poisoning is very high in both ways. After oral use, contacting with moisture, and reacting with gastric acid, aluminum phosphide produces phosphine gas that is easily absorbed through the digestive system. Phosphine gas plays an important role in toxicity (7). The exact mechanism of the effects of aluminum phosphide in humans is not clear, and it seems that intense cell damage is the aluminum phosphide’s main mechanism of the action, which does not include only a single organ, rather results in a multi-organ failure (3).

The main symptoms of the disease include shock (hypotension, tachycardia, tachypnea, etc.), headache, dizziness, nausea, vomiting, and severe metabolic acidosis; the mentioned symptoms result in the subsequent involvement of vital organs including heart, brain, lungs, kidneys, and liver, and finally lead to death (8). In order to overcome acidosis, there is a need for continuous infusion of sodium bicarbonate. Effecting on the cardiovascular system, acidosis usually makes it difficult and sometimes impossible to recover a patient. Controlling metabolic acidosis in patients can be very effective in the prognosis of their treatment (9).

Hydroxyethyl starch is a colloid solution that is widely used to increase plasma volume in shocks induced by trauma or general infections. Given the fact that the benefit of using hydroxyethyl starch helps a patient survive was reported for the first time in a case report in Iran (10), and various studies have reported the positive effects of hydroxyethyl starch therapy in controlling shock. This study aimed to investigate the effects of the administration of this intravenous solution on the prognosis of a group of patients with aluminum phosphide poisoning who referred to poisoning ICU in Baharloo Hospital from April 2012 to April 2015.

## Methods

This study was conducted as a randomized clinical trial which was approved by Ethics Committee of Tehran University of Medical Sciences on 19 February 2017, (ethical code: IR.TUMS.VCR.REC.1395.1741). The study population was consisted of 60 Iranian patients with aluminum phosphide poisoning who were admitted to the poisoning ICU in Baharlo Hospital from April 2012 to April 2014. Patients were entered into the study after obtaining the informed consent.

 A total of 60 poisoned patients were enrolled in the study, of whom 30 patients were randomly allocated to the starch treatment group and 30 patients to the normal saline group. The study was conducted on patients who were diagnosed with aluminum phosphide poisoning (based on their history and clinical symptoms), experienced metabolic acidosis (bicarbonate of less than 14), and had systolic blood pressure of less than 90 mmHg during the first six hours of admission. Following the diagnosis of poisoning by a physician, starch (6%) is routinely administered in the hospital; thus, the records of patients treated with starch was reviewed and compared with the records of those who received normal saline in the hospital.

Patient's demographic data (age, sex, etc.) and data on the disease including base excess before and after the solution therapy, systolic blood pressure, serum bicarbonate, arterial pH in the first 24 hours of admission, and arterial blood gas (ABG) before the injection and after the first hour were recorded. After collecting the required information, SPSS21 software was used to analyze the data. Independent t-test was used to compare the two groups. In addition, paired t-test was used to compare the values obtained before and after the treatment. 

Moreover, tables and statistical indicator central tendency and dispersion were used for descriptive analysis of data. It is worth noting that, since the sample size in the study was not very large, non-parametric tests were used for non-normal data.

## Results

The mean age of the patients (± standard deviation) in the two groups of starch and normal saline was 25.13±9 years and 26.13±9.50 years, respectively. In the normal saline group, 50% were males and 50% were females, and in the starch group, 43.3% were males and 56.7% were females. There was no statistically significant difference between the two groups in terms of the mean age and sex.

The mean systolic blood pressure in the normal saline group and starch group at the beginning of the study was 88.30 and 89.10 mmHg, respectively, and there was not statistically significant difference between the two groups. The comparison of the mean of arterial base excess at the beginning of the study showed no significant difference between the two groups in terms of the mean arterial base excess and / or arterial PH. Thus, at the beginning of the study, the two groups were similar to each other in terms of the mean arterial base excess.

**Results of the assessment of arterial PH: **The results of arterial pH measurement are presented in table 1. As shown, the arterial pH value in the starch group changed from 7.12 before the treatment to 7.25 after the treatment, and in the normal saline group changed from 7.09 before the treatment to 7.27 after the treatment. In fact, arterial pH in the starch group and normal saline group increased by 0.13 and 0.18, respectively, and the difference between the two groups was not statistically significant. Comparison of the values of arterial pH between males and females showed that the results obtained for males and females did not differ with the overall results. 

Although there was a significant difference between the values measured before and after the treatment in males and females, the difference between the two groups of the starch and normal saline was not significant (Table 2).

**Results of the assessment of arterial blood base excess: **The results of the measurement of arterial blood base excess showed that, base excess in the starch group changed from 12.71 before the treatment to 19.12 after the treatment, and in the normal saline group it changed from 12.62 before the treatment to 18.01 after the treatment. Changes in base excess before and after the treatment in the starch group and normal saline group was 6.41 and 5.39, respectively, and the difference between the two groups was not statistically significant. The results of comparing arterial base excess in the two groups are presented in table 3.Comparison of the values of arterial base excess between males and females showed that the results obtained for males and females did not differ with the overall results. Although there was a significant difference between the values measured before and after the treatment in males and females, the difference between the two groups of the starch and normal saline was not significant (table 4).

The mean values measured before and after the intervention were analyzed in each group separately using paired t-test, and the results showed that the changes in mean values were significant in both groups and the p-value in each group was less than 0.05. It indicates that both normal saline and starch are effective in treating acidosis, but one cannot say which of them has a better performance.

Of all the studied patients, 17 (56.7%) patients in the starch group and 17 patients in the normal saline group (out of 30 persons in each group) died; thus, the number of people in the two groups is exactly the same.

**Table 1:** Comparison of the mean arterial pH between the starch group and normal saline group

**Treatment group**	**Before the treatment**	**After the treatment**	**p- value **	**Mean difference**
Normal saline	7.09 (0.11)	7.27 (0.14)	< 0001	0.18 (0.08)
Hydroxyethyl starch	7.12 (0.12)	7.25 (0.18)	0.001	0.13 (0.09)
p- value **	0.390	0.758		0.091

The numbers represent mean and standard deviation.

** P-value of the comparison between the two groups.

**Table 2:** Comparison of the mean arterial pH between the starch group and normal saline group by sex

	**Treatment group**	**Males**				**Females**			
		**Before the treatment**	**After the treatment**	**p- value **	**Mean difference**	**Before the treatment**	**After the treatment**	**p- value **	**Mean difference**
	Normal saline	7.09 (0.08)	7.27 (0.13)	<0.0001	0.14 (0.08)	7.08 (0.14)	0.16 (7.27)	<0.0001	0.18 (0.09)
	Hydroxyethyl starch	7.12 (0.12)	7.26 (0.19)	<0.0001	0.17 (0.07)	7.11 (0.13)	7.24 (0.17)	<0.0001	0.13 (0.11)
	p- value **	0.441	0.931		0.227	0.684	0.703		0.228

The numbers represent mean and standard deviation.

** P-value of the comparison between the two groups.

**Table 3:** Comparison of the mean arterial base excess between the starch group and normal saline group

Treatment group	Before the treatment	After the treatment	p- value	Mean difference
Normal saline	12.62 (1.52)	18.01 (6.32)	< 0.0001	5.39 (5.45)
Hydroxyethyl starch	12.71 (1.35)	19.12 (9.29)	0.018	6.41 (8.82)
p- value **	0.154	0.569		0.218

The numbers represent mean and standard deviation.

** P-value of the comparison between the two groups

**Table 4:** Comparison of the mean arterial pH between the starch group and normal saline group by sex

	**treatment group**	**Males**				**Females**			
		**Before the treatment**	**After the treatment**	**p-value **	**Mean difference**	**Before the treatment**	**After the treatment**	**p- value **	**Mean difference**
	Normal saline	11.58 (1.39)	15.44 (6.40)	<0.0001	3.85 (5.76)	13.68 (0.44)	20.59 (4.71)	0.001	6.90 (4.44)
	Hydroxyethyl starch	12.67 (1.37)	17.35 (6.76)	0.007	4.67 (6.24)	12.75 (1.47)	21.45(12.31)	0.021	8.69(11.76)
	p- value **	0.065	0.420		0.703	0.056	0.804		0.589

The numbers represent mean and standard deviation.

** P-value of the comparison between the two groups

## Discussion

To our knowledge, this study is the first study conducted to evaluate the effect of hydroxyethyl starch on the treatment of severe acidosis and to compare its effect with that of normal saline in a group of patients with aluminum phosphide poisoning. As the results showed, both normal saline and hydroxyethyl starch were effective in improving acidosis and resulted in a significant statistical difference in the values observed before and after the treatment. However, after comparing the changes in the two groups, it was found that the two groups did not have a statistically significant difference. In fact, as compared with the normal saline, starch caused more changes in acidosis after the treatment, nevertheless, the difference was not statistically significant.

The lack of significant differences between the two groups can be attributed to two factors. First, it may be due to the lack of a real significant difference between normal saline and starch in terms of their effects in treating acidosis in patients with poisoning. Second, there might have been a significant difference between the two groups, but in the present study it was not observed due to the small sample size; in other words, the power of the study was not high enough to show a significant difference between the two groups. Therefore, it is necessary to conduct further studies with a larger sample size to confirm the results of the present study. This topic was not investigated in a clinical trial before this study; nevertheless, in a case study conducted by Marashi et al. in 2016, it has been shown that treatment with a starch in a patient with aluminum phosphide poisoning had an interesting result. As they reported, six hours after the onset of treatment with starch in the patient, acidosis was controlled and eventually the patient survived. The authors note that, as a fact, the exact mechanism of rice tablet poisoning is not known. Cytotoxicity induced by mitochondria toxicity, inhibition of cytochrome C oxidase, formation of free radicals, lipid peroxidation and subsequent cellular damage, and even the reduction of cellular glutathione are possible mechanisms of this type of poisoning (4, 5, 8, 11, 12). For many years, gastric lavage, potassium permanganate, and activated charcoal have been used as the major and primary treatment for rice tablet poisoning in emergency wards. However, the oxidation of phosphine gas and the absorption of aluminum phosphide in the gastrointestinal tract cause acute toxicity and also complications such as hemodynamic defects which lead to the death of patients. Therefore, there are studies that reject routine therapies (13, 14). Hydroxyethyl starch, as an artificial colloid, is the most common colloid used in the intensive care unit. The length of time this fluid can remain in the intramuscular space is significant. This fluid can reduce the leakage of fluid and albumin from injured endothelial cells and reduce serum lactate levels (15). 

Various studies have shown that hydroxyethyl starch can act as a good fluid for controlling acidosis in patients who need urgent treatment, especially after surgery (16-18). Hence, taking into account the high efficacy of this substance as well as its insignificant side effects, it can be recommended as a good alternative for controlling acidosis in patients with rice tablet poisoning. It seems that the decrease in blood pressure and the increase in vascular endothelial leakage and severe acidosis during this poisoning are the main causes of poor prognosis and low rate of survival in these patients. 

Hydroxyethyl starch seems to be a good alternative for reducing the symptoms in the patients, thus, it is necessary to conduct further studies to prove this hypothesis. As one of the limitations of this study, it was not possible for us to measure sodium and potassium and to compare them between the two groups. Given that sodium and potassium equilibrium can affect the acidic environment, the mentioned limitation could affect our results.

In conclusion, overall, the results of the present study show that starch is at least as effective as normal saline in treating acidosis in patients poisoned with rice tablets and can be used instead of normal saline, but we could not confirm which of them has a better performance. It may be needed to conduct further studies, with a larger sample size.
